# Photochemistry and Antioxidative Capacity of Female and Male *Taxus baccata* L. Acclimated to Different Nutritional Environments

**DOI:** 10.3389/fpls.2018.00742

**Published:** 2018-06-05

**Authors:** Piotr Robakowski, Emilia Pers-Kamczyc, Ewelina Ratajczak, Peter A. Thomas, Zi-Piao Ye, Mariola Rabska, Grzegorz Iszkuło

**Affiliations:** ^1^Department of Forestry, Poznan University of Life Sciences, Poznań, Poland; ^2^Institute of Dendrology, Polish Academy of Sciences, Kórnik, Poland; ^3^School of Life Sciences, Keele University, Keele, United Kingdom; ^4^College of Maths & Physics, Jinggangshan University, Ji'an, China; ^5^Faculty of Biological Sciences, University of Zielona Góra, Zielona Góra, Poland

**Keywords:** apparent electron transportation rate, carotenoids, chlorophyll *a* fluorescence, dioecious plants, antioxidants, English yew, photosynthesis

## Abstract

In dioecious woody plants, females often make a greater reproductive effort than male individuals at the cost of lower growth rate. We hypothesized that a greater reproductive effort of female compared with male *Taxus baccata* individuals would be associated with lower female photochemical capacity and higher activity of antioxidant enzymes. Differences between the genders would change seasonally and would be more remarkable under nutrient deficiency. Electron transport rate (ETR_max_), saturation photosynthetic photon flux corresponding to maximum electron transport rate (PPF_sat_), quantum yield of PSII photochemistry at PPF_sat_ (Φ_PPFsat_), and chlorophyll *a* fluorescence and activity of antioxidant enzymes were determined in needles of *T. baccata* female and male individuals growing in the experiment with or without fertilization. The effects of seasonal changes and fertilization treatment on photochemical parameters, photosynthetic pigments concentration, and antioxidant enzymes were more pronounced than the effects of between-sexes differences in reproductive efforts. Results showed that photosynthetic capacity expressed as ETR_max_ and Φ_PPFsat_ and photosynthetic pigments concentrations decreased and non-photochemical quenching of fluorescence (NPQ) increased under nutrient deficiency. Fertilized individuals were less sensitive to photoinhibition than non-fertilized ones. *T. baccata* female and male individuals did not differ in photochemical capacity, but females showed higher maximum quantum yield of PSII photochemistry (F_v_/F_m_) than males. The activity of guaiacol peroxidase (POX) was also higher in female than in male needles. We concluded that larger *T*. *baccata* female reproductive effort compared with males was not at the cost of photochemical capacity, but to some extent it could be due to between-sexes differences in ability to protect the photosynthetic apparatus against photoinhibition with antioxidants.

## Introduction

The main advantages of dioecious species over hermaphroditic are complete exclusion of the risk of self-pollination (Darwin, 1892; Charlesworth and Charlesworth, 1978) and optimization of resource allocation in both male and female functions (Maynard-Smith, 1978; Charnov, 1982). However, this mating system is relatively rare; only 6% of angiosperms are dioecious (Renner and Ricklefs, 1995; Renner, 2014). One of the reasons for the small number of dioecious species is reduced seed dispersal because only half of individuals (females) produces diaspores (Heilbuth et al., 2001). Moreover, females usually invest in greater reproductive effort over a longer period compared to males (Garbarino et al., 2015; Vessella et al., 2015; Matsushita et al., 2016). In woody dioecious species this greater investment by females in reproduction effort is often linked to lower growth rates than in males supporting the costs of reproduction hypothesis (e.g., Obeso, 2002; Leigh et al., 2006). Sexual differences in reproductive effort, stress response or different environment requirements may cause dioecious species to be more sensitive to environmental changes (Rozas et al., 2009; Chen et al., 2014). Sex-related differences frequently arise or are greater under stressful conditions (such as drought and thermal stress), indicating a greater sensitivity of females to poorer habitat conditions (Zhang et al., 2010; Iszkuło et al., 2011b; Zhao et al., 2011; DeSoto et al., 2016). Females have higher reproductive effort and are adapted to higher-resource microsites compared with males (Vessella et al., 2015). A consequence is that environmental stress caused by less-than-optimal light, nutrition, or water conditions often favors maleness (reviewed by Korpelainen, 2007).

Females often have a greater intensity of gas exchange in comparison to males (Dawson and Ehleringer, 1993; Obeso, 2002; Montesinos et al., 2012) and increase photosynthesis by increasing leaf area (Wallace and Rundel, 1979; Meagher, 1992; Kohorn, 1994). Nevertheless, this is not clear whether between-sexes differences in net CO_2_ assimilation rates result from dark photosynthetic processes or photochemistry. We suggest, therefore, that differences in photochemical capacity between sexes would be related to their reproductive efforts, in agreement with the optimal resource use hypothesis of Bloom et al. (1985). However, the cost-benefit balance of sex-related differences in photosynthesis and photoprotection in leaves of dioecious plants has been poorly studied to date (Juvany et al., 2014). Previous studies indicate that males and females might not differ in maximum quantum yield of photosystem II (PSII) photochemistry (F_v_/F_m_) but males had enhanced photoprotection. Nevertheless, differences in photochemical efficiency and capacity between males and females under nutrient deficiency are not fully understood (Juvany et al., 2014). Photosynthesis is the main source of damaging reactive oxygen species (ROS) (Foyer and Shigeoka, 2011). Therefore, enzymatic and non-enzymatic antioxidant systems are required to maintain intracellular ROS pools at low levels (Ratajczak et al., 2015) and mitigate potentially harmful reactions caused by enhanced oxidative load (Foyer and Shigeoka, 2011). Enzymatic antioxidant systems in photosynthetic processes involve the action of superoxide dismutase, peroxidases, catalases, and reductases (Ding et al., 2016). Superoxide dismutase (SOD) is involved in the detoxification of superoxide anion (${\text{O}}_{2}^{\cdot -}$) resulting in molecular oxygen (O_2_) or the formation of hydrogen peroxide (H_2_O_2_). The latter is subsequently reduced by catalase and peroxidases to water with the generation of monodehydroascorbate. These antioxidants not only have specific protective functions in photosynthesis, but are also associated with redox signaling during this process (Foyer and Noctor, 2011; Benzarti et al., 2012). It is expected that female plants would show higher levels of antioxidants because of their higher metabolic activity, in particular, their higher activity of photosynthesis. The metabolic rate increases steeply in relation to reproductive effort in females. This increase in metabolism results in higher production of ROS, which may initiate oxidative stress (Zinta et al., 2016). Therefore, a highly active antioxidant system is necessary to maintain homeostasis in the cells (Foyer and Shigeoka, 2011). Reduction of oxidative stress may underlie the trade-off between reproduction and survival of females.

*Taxus baccata* is a suitable model for studying dioecious species because males and females are known to respond differently to environmental conditions. Males of *T. baccata* grow taller (Iszkuło et al., 2009), show greater radial growth than females after the beginning of sexual maturity (Cedro and Iszkuło, 2011; Iszkuło et al., 2011a), and females have lower N concentration in needles, especially when undergoing intensive shoot elongation and radial growth (Nowak-Dyjeta et al., 2017). *T*. *baccata* is threatened by extinction due to its low tolerance of a range of environmental stresses and its intensive exploitation in the past (Thomas and Polwart, 2003; Iszkuło et al., 2016; Kýpetová et al., 2018).

Our overarching hypothesis was that greater reproductive effort of *T*. *baccata* females compared with males would be reflected in their (1) lower photosynthetic capacity presented by lower quantum yield of PSII photochemistry at the saturation level of PPF (Φ_PPFsat_), lower F_v_/F_m_ and lower apparent maximum electron transport rate (ETR_max_), (2) higher energy losses as heat assessed with non-photochemical quenching of fluorescence (NPQ), and (3) higher activity of antioxidant enzymes. An additional hypothesis was that fertilization effects on photochemistry and antioxidant activity would be more pronounced than differences between sexes. Moreover, we suggest that the differences between male and female individuals would be reduced under higher nutrient availability.

## Materials and methods

### Experimental design

The experiment was conducted at the Institute of Dendrology, Polish Academy of Sciences in Kornik, Poland. Rooted shoots of *T*. *baccata* were used in the experiment. In 2012, fifty shoots were collected and rooted from 20 trees (10 males and 10 females) of *T*. *baccata* growing in the Kornik Arboretum (two genders x 10 individuals x 50 shoots = 1,000 plants). Cuttings of similar size were taken from the middle part of each crown, growing in similar, partially-shaded light conditions. Individuals were grown in 5-liter pots under 2-m-high scaffolding with shading net to produce a 50% reduction in full sunlight. The degree of light reduction was confirmed by measurements of relative photosynthetic photon flux density using a line quantum sensor (Apogee Inc.) following the methods of Messier and Puttonen (1995). The soil for the pots was obtained from a natural broadleaved forest, similar to the typical habitat of the study species. Soil (10% of the soil volume) was added from a stand of *T*. *baccata* to ensure natural mycorrhizal inoculation. In March 2013, seedlings were randomly divided into two blocks containing both genders, and then within each block two fertilization treatments were established. The fertilized group of seedlings received 6 g per liter of Osmocote Exact 5-6 M (ICL, Israel) in March 2014 and 2015, whereas non-fertilized seedlings were grown without any fertilizer. The fertilizer contained 15% N, 9% P, 12% K, 2.5% MgO, and microelements. Needles were sampled over the two following years every 4 months: (1) in March, at the end of the flowering period before the beginning of growth; (2) in June when mass allocation to aboveground organs reaches a maximum, at the end of height growth; (3) in September, at the end of the mass allocation to roots and other parts of a plant but while females are investing resources in maturing seeds and arils; (4) in December, when plants are dormant. At each sampling point, 1-year needles from 24 seedlings were used for all analysis (3 seedlings x 2 fertilization treatments x 2 genders x 2 blocks). Needles were taken from the same shoot from the top of individuals for all analyses.

### Meteorological conditions

During the experiment, air temperature and relative air humidity were recorded each hour using four EL-USB-2+ data loggers (EasyLog, Inc.). Monthly mean (T_mean_), monthly minimum (T_min_), and maximum (T_max_) values of air temperatures and monthly mean air relative humidity (RH) over the sampling period are shown in Table 1. The difference between the highest and lowest values of T_mean_ in June 2014 and December 2015 was 20.3°C. During the experiment, T_max_ was in June 2015 and T_min_ was in December 2015 (T_max_−T_min_ = 58°C). Mean air relative humidity was highest in January 2015 and it attained the minimum in June 2014 (RH_max_−RH_min_ = 33.9%).

**Table 1:** Meteorological conditions at the time of *T. baccata* needle sampling.

**Year**	**Month**	**Monthly temperature (°C)**			**Relative air humidity (%)**
		**Mean**	**Minimum**	**Maximum**	
2014	March	7.5 ± 0.3	−6.0	29.5	76.5 ± 0.8
	June	18.4 ± 0.3	4.5	40.5	68.0 ± 0.8
	September	16.7 ± 0.3	0.0	36.0	77.4 ± 0.8
	December	1.8 ± 0.1	−8.0	14.5	91.9 ± 0.4
2015	March	5.8 ± 0.2	−7.0	25.5	75.1 ± 0.7
	June	18.1 ± 0.3	4.0	42.5	68.3 ± 0.9
	September	15.1 ± 0.3	0.5	40.5	73.2 ± 0.7
	December	−1.9 ± 0.2	−15.5	11.0	90.6 ± 0.4

*Data are monthly temperatures (mean, minimum, and maximum) and monthly means of air relative humidity (±SE) are given*.

### Chlorophyll *a* fluorescence

Chlorophyll *a* fluorescence was measured in needles using a Fluorescence Monitoring System (FMS 2, Hansatech, Norfolk, UK) operating in an online mode. Collected 1-year needles were wrapped in moist paper, enclosed in Eppendorf tubes, and brought to the laboratory. Prior to fluorescence measurements, needles were dark adapted for 30 min at air temperature around 22°C (21–23°C) in the laboratory, arranged tightly side-by-side, stuck on self-adhesive transparent tape to fill the entire aperture of the factory-provided clip. The mean air temperature of fluorescence measurements was 22°C (21–24°C, min.-max.), monitored using a thermocouple installed in the leaf clip. Using a light-tight chamber containing a light source inserted onto the leaf clip, the needles were exposed to modulated light at 0.05 μmol quanta m^−2^ s^−1^. After reading minimum fluorescence F_0_, a saturating 0.7 s pulse of light (PPF = 15.3 mmol m^−2^ s^−1^) was delivered to induce a maximum fluorescence (F_m_). Maximum quantum yield of PSII photochemistry was calculated as F_v_/F_m_, where F_v_ = F_m_-F_0_.

Subsequently, to generate light response curves of PSII quantum yield (Φ_PSII_) needles in the clip were illuminated with actinic light using an inbuilt halogen lamp. The intensity of actinic light corresponding to values indicated by the software was measured prior to the experiment using a light sensor inserted in the leaf-clip in the position of the needles. Up to 12 levels of actinic light of increasing intensity were used, and for each level, after a stable steady state fluorescence (F_s_) was reached, 0.7 s saturating pulse was delivered and maximum light-adapted fluorescence (F'_m_) was determined. Quantum yield of PSII was calculated as: Φ_PSII_ = (F'_m_–F_s_)/F'_m_ (Genty et al., 1989). At each actinic light level, non-photochemical quenching of fluorescence (NPQ) was calculated as: NPQ = (F_m_−F'_m_)/F'_m_ (Maxwell and Johnson, 2000). The course of fluorescence and all the measured parameters were constantly monitored to ensure stable F_s_ values after changing actinic illumination levels before a saturating pulse was applied. Usually, stabilization time of F_s_ took 1.5–3.5 min.

For each light level, the apparent rates of photosynthetic electron transport (ETR) were calculated as: ETR = α ^*^ Φ_PSII_^*^ PPF^*^0.5 (α—needle absorptance, Maxwell and Johnson, 2000). Assumptions were made that the excitation energy is partitioned equally between the two photosystems (hence the factor of 0.5; Maxwell and Johnson, 2000). Leaf absorptance may differ among plants depending on species and adaptation to microclimate conditions. In our study, it was calculated using the model by Evans (1993), which is based on total chlorophyll content in leaf.

#### Determination of cardinal points of light response curves

The maximum apparent rate of photosynthetic electron transport of PSII (ETR_max_) and the saturation level of photosynthetic photon flux density (PPF_sat_) in Equation (1) were derived by fitting the functions of Ye et al. (2013):

(1) $$\begin{array}{l} \text{ETR}=\alpha \frac{1-\beta \text{PPF}}{1+\gamma \text{PPF}}\text{PPF}, \end{array}$$

where α is the initial slope, β is the extent of dynamic down-regulation of PSII, and γ is defined as a saturation term of light response curve for photosynthetic electron transport rate (ETR-PPF), PPF is photosynthetic photon flux.

PPF_sat_ is calculated from the Equation (2):

(2) $$\begin{array}{l} \text{PP}{\text{F}}_{\text{sat}}\;=\frac{\sqrt{\left(\beta +\gamma \right)/\beta }-1}{\gamma }, \end{array}$$

ETR_max_ has been defined as ETR at PPF_sat_ and is derived from the Equation (3):

(3) $$\begin{array}{l} {\text{ETR}}_{\text{max}}=\alpha {\left(\frac{\sqrt{\beta +\gamma }-\sqrt{\beta }}{\gamma }\right)}^{2}, \end{array}$$

To estimate quantum yield of PSII photochemistry at saturating PPF (Φ_PPFsat_), the exponential rise to maximum function was fitted to light curves of Φ_PSII_ (Rascher et al., 2000; Robakowski, 2005) as in Equation (4).

(4) $$\begin{array}{l} \Phi_{\text{PPFsat}}\;=\text{m}+{\text{ae}}^{-\text{bPPFsat}}+{\text{ce}}^{-\text{dPPFsat}}, \end{array}$$

where a, b, c, d, m are independent parameters.

The PPF vs. NPQ curves were fitted with the exponential function in the Equation (5):

(5) $$\begin{array}{l} \text{NPQ }=\text{m}+\text{a}\left(1-{\text{e}}^{\left(-\text{bPPF}\right)}\right), \end{array}$$

Light curves of NPQ are considered to rise to infinity (Maxwell and Johnson, 2000). Therefore, NPQ was calculated at the arbitrary value of 345 μmol m^−2^ s^−1^ of actinic light (NPQ_345_) and used in estimating the effects of time, fertilization, and sex on energy losses as heat. Light curves of Φ_PSII_, ETR, and NPQ were fitted with the above functions to derive all parameters using the non-linear estimation of Levenberg-Marquardt in Statistica 13.1 (Tulsa, USA).

### Chlorophyll and total carotenoids in needles

The one-year needles (40–50 mg of fresh weight) were used for spectrophotometric analyses of chlorophyll and total carotenoids contents. They were cut into 2 mm pieces and incubated in 5 ml of 100% dimethylsulfoxide (DMSO) saturated with CaCO_3_ to avoid pheophytization, in a water bath at 60°C until the solution became translucent (~5 h). The absorbance of the extract was measured at 665, 648, and 470 nm. Chlorophyll *a, b*, and total carotenoids contents were calculated using the formulae given by Barnes et al. (1992).

### Total enzyme activity

The whole one-year needles were used for analysis of all enzymes. All extraction procedures were conducted at 4°C. Samples were ground in liquid nitrogen and homogenized in 50 mM sodium phosphate buffer, pH 7.0, containing 0.2 mM EDTA, and 20% polyvinylpolypyrrolidone (PVPP) and were incubated for 1 h in the cold. The homogenates were filtered through two layers of cheesecloth and centrifuged at 4°C at 20,000 g for 20 min. The supernatant was desalted on a Sephadex G25 (Sigma-Aldrich) standard column according to Helmerhorst and Stokes (1980). Analysis of enzyme activity was performed in the cytosolic fraction.

The superoxide dismutase (SOD, EC. 1.15.11) activity was determined using the method of Giannopolitis and Ries (1977) by measuring its ability to inhibit the photochemical reduction of 4-nitro blue tetrazolium chloride (NBT), as described in Pukacka and Pukacki (2000). One unit (1U) of SOD activity was defined as the amount of enzyme required to cause 50% inhibition of the rate of NBT reduction (U mg^−1^ protein). The 3 ml reaction mixture contained 50 mM phosphate buffer (pH 7.8), 1.3 mM riboflavin, 0.1 mM dithiothreitol (DTT), 63 mM NBT, and 50 μl enzyme extract. The mixture was illuminated in glass test tubes. A non-irradiated reaction mixture served as a control.

Guaiacol peroxidase and catalase were determined according to Chance and Maehly (1955). Total guaiacol peroxidase (POX, EC. 1.11.1.7) activity was measured by the oxidation of guaiacol at 470 nm (ε = 26.6 mM^−1^ cm^−1^). The reaction mixture contained 1 ml of 100 mM potassium phosphate buffer at pH 7.0, 1 ml of 0.089 mM guaiacol, 1 ml of 200 mM H_2_O_2_, and 50 μl of enzyme extract. The reaction was initiated by adding H_2_O_2_. Controls were made for the background absorbance at 470 nm, without H_2_O_2_ (one control) and without guaiacol (second control). The reaction was initiated by adding H_2_O_2_. POX activity was expressed as nkat min^−1^ mg. protein^−1^.

For catalase (CAT, EC 1.11.1.6) activity, the reaction mixture contained 1 ml of 0.1 M phosphate buffer pH 7.0, 1 ml of 30 mM H_2_O_2_, and 50 μl of enzyme extract. The catalase activity was determined by the decrease in absorbance at 240 nm. The reaction was initiated by adding H_2_O_2_. Controls were made for the background absorbance at 240 nm, without H_2_O_2_ (one control) and without enzyme extract (second control). CAT activity was expressed as mmol H_2_O_2_ min^−1^ mg. protein^−1^.

Cytosolic ascorbate peroxidase (APX, EC.1.11.1.11) activity was measured by following the decrease in absorbance at 290 nm due to ascorbic acid (ASA) oxidation for 5–10 min. according to Nakano and Asada (1981). The reaction mixture contained: 1 ml of 0.68 mM ASA, 0.1 mM EDTA in 0.1 M phosphate buffer at pH 7.0, 1 ml of 4 mM H_2_O_2_, and 50 μl of the enzyme extract. The reaction was initiated by adding H_2_O_2_. A control was made for the low, non-enzymatic oxidation of ASA by H_2_O_2_. APX activity was expressed as nmol ASA min^−1^ mg. protein^−1^.

The protein content of crude enzyme extracts was estimated according to Bradford (1976), using bovine serum albumin as a standard.

### Statistics

Prior to analyses, all data were tested for normality using the Shapiro-Wilk's test and homogeneity of variance with the Levene's test. Results were analyzed with the full-factorial three way analysis of variance with gender, fertilization group, and time of sampling as the sources of fixed effects and with blocks as the source of random effect. When interactions occurred, Student *t*-test was applied to test differences between means. Means were considered to differ statistically at *P* < 0.05. Data were presented as means with standard errors (SE). All analyses were done using JMP 12 software (SAS Institute Inc., Cary, NC, 1989-2007). Figures were prepared with SigmaPlot v. 14 (Systat Software, Inc., San Jose California USA).

## Results

### Light curves of fluorescence

**Figure 1:**
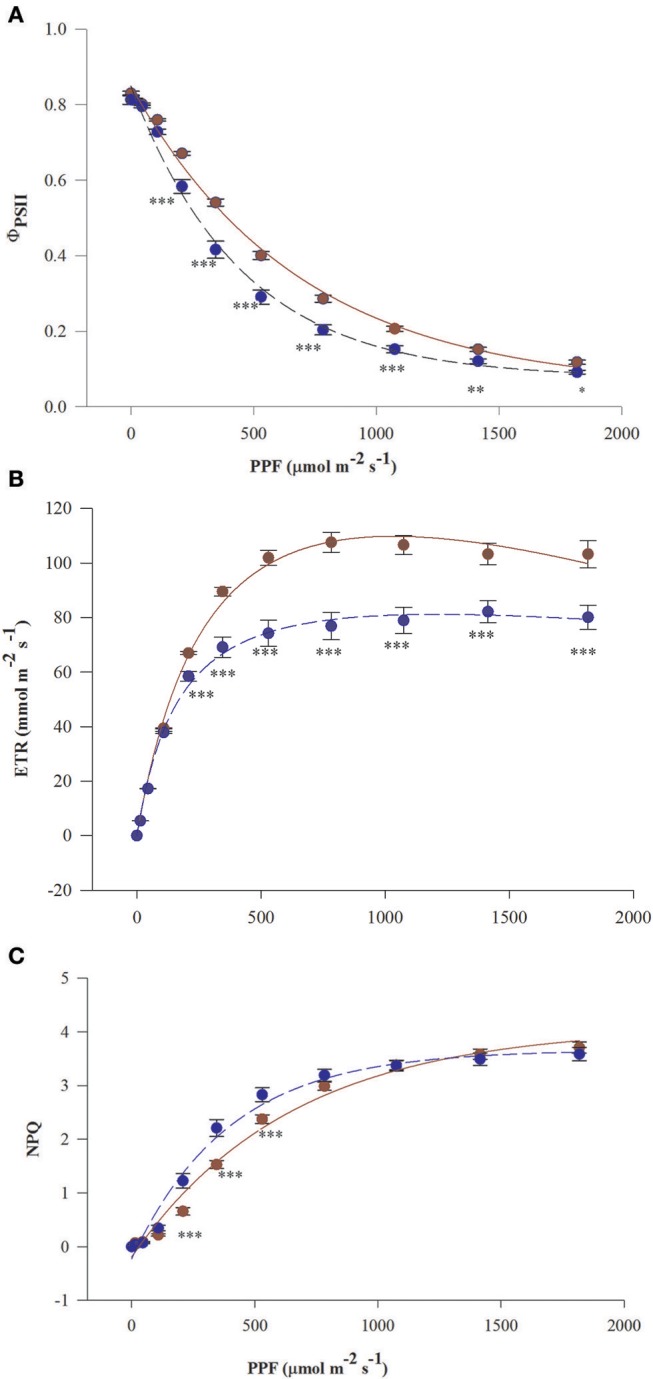
Light response curves of needles of *T. baccata* individuals growing with (brown solid line) or without fertilization (dark blue dotted line): **(A)** Photosystem II (PSII) quantum yield (Φ_PSII_), **(B)** Apparent electron transfer rate (ETR), and **(C)** Non-photochemical quenching of fluorescence (NPQ) vs. photosynthetic photon flux (PPF). The fluorescence measurements were conducted in June 2015. The equation used for non-linear fitting together were as follows: **(A)** $\Phi_{\text{PPFsat}}\;=\text{m}+{\text{ae}}^{-\text{bPPFsat}}+\text{c}{\text{e}}^{-\text{dPPFsat}}$, **(B)** $\text{ETR}=\alpha \frac{1-\text{βPPF}}{1+\text{γPPF}}\text{PPF},$ **(C)** NPQ = m+a(1−e^(−bPPF)^). The values of adjusted coefficients of determination and probability were described by $R_{\text{adj}}^{2}$ ≥ 0.98 with *P* < 0.001 for all used equations. Data are means with standard errors (SE, *n* = 12). Asterisks indicate statistical differences between means at ^*^0.05 > *P* ≥ 0.01, ^**^0.01 > *P* ≥ 0.001, *P* < 0.001^***^.

Curves of Φ_PSII_ and ETR vs. PPF in needles of fertilized *T*. *baccata* individuals run above the curves in needles of non-fertilized individuals except for the initial part of the curves at low PPF (Figures 1A,B). Significant differences in mean values of Φ_PSII_, ETR, and NPQ between fertilization treatments were observed beginning from 208 μmol m^−2^ s^−1^ of fluorescence induction light. The photosynthetic capacity of non-fertilized plants estimated by the mean value of ETR_max_ was about 30% lower compared with fertilized ones. Non-fertilized individuals lost more needle absorbed energy as heat reflecting the higher NPQ values in non-fertilized individuals except for when NPQ was low (up to 208 μmol m^−2^ s^−1^) and high (above 800 μmol m^−2^ s^−1^) PPF of induction light (Figure 1C). There were not significant differences between the sexes in Φ_PSII_, ETR, and NPQ vs. PPF.

### Time-course of photochemical parameters

**Figure 2:**
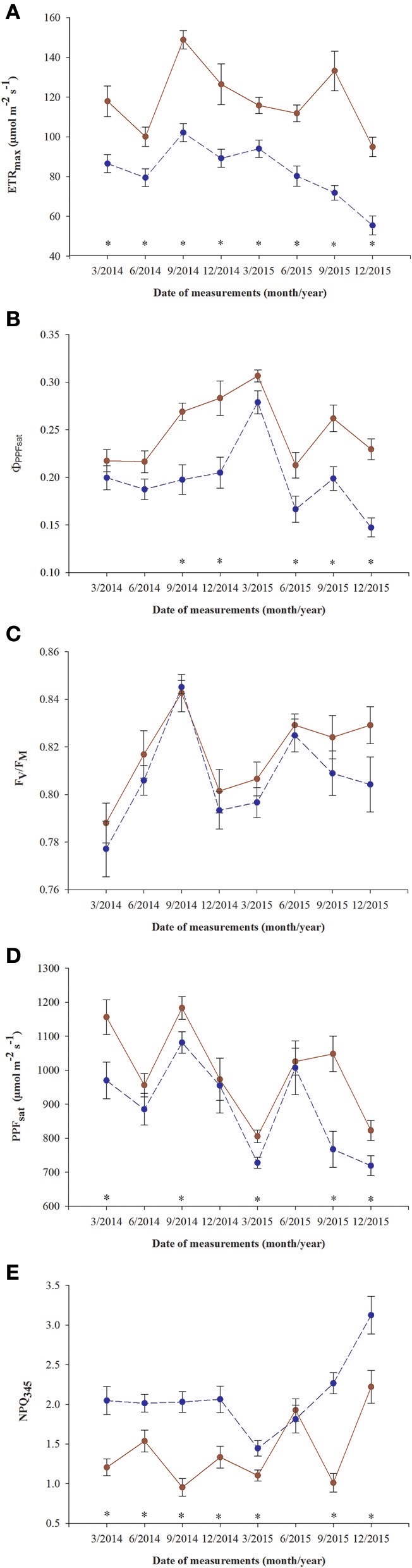
Changes in time in photosynthetic parameters in needles of *T. baccata* individuals growing with (brown solid line) or without fertilization (dark blue dotted line): **(A)** Apparent maximum electron transport rate (ETR_max_); **(B)** Quantum yield of PSII photochemistry at the saturation value of photosynthetic photon flux (Φ_PPFsat_); **(C)** Maximum quantum yield of PSII photochemistry (F_v_/F_m_); **(D)** Saturation photosynthetic photon flux corresponding to maximum electron transport rate (PPF_sat_); and **(E)** Non-photochemical quenching of fluorescence at PPF = 345 μmol m^−2^ s^−1^ (NPQ_345_). Data are means with standard errors (SE, *n* = 12). Asterisks indicate statistical differences between means at *P* < 0.05.

**Figure 3:**
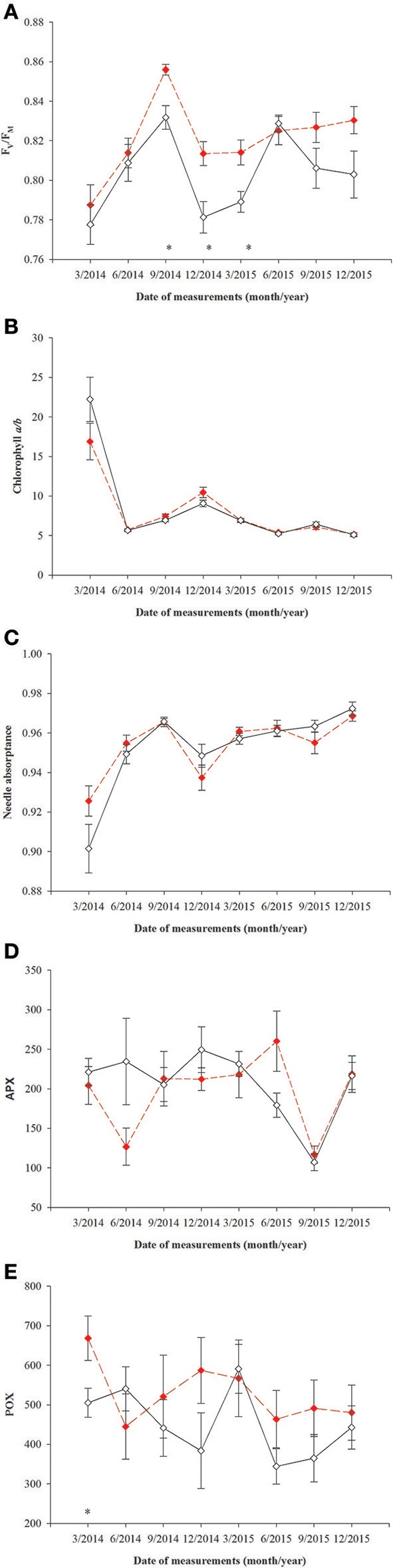
Changes with time in photochemistry in needles of *T. baccata* male (black solid line) and female (red dotted line) individuals: **(A)** Maximum quantum yield of PSII photochemistry (F_v_/F_m_); **(B)** Chlorophyll *a/b* ratio; **(C)** Needle absorptance (α); **(D)** ascorbate peroxidase (1 nmol ASA min^−1^ mg. protein^−1^, APX); and **(E)** guaiacol peroxidase (nkat min^−1^ mg. protein^−1^, POX). Data are means with standard errors (SE, *n* = 12). Asterisks indicate statistical differences between means with *P* < 0.05.

All parameters based on chlorophyll *a* fluorescence underwent significant seasonal changes and differed significantly between fertilization treatments (Table 2, Supplementary file—Table 1). Generally, fertilized individuals had higher values of ETR_max_, Φ_PPFsat_, PPT_sat_, and F_v_/F_m_ (Figures 2A–D) and lower values of NPQ_345_ (Figure 2E) compared with non-fertilized individuals. The mean values±SE of ETR_max_ for fertilized and non-fertilized individuals were 121 ± 3 and 83 ± 2 μmol m^−2^ s^−1^, respectively. The differences in this parameter between fertilization treatments were significant before the appearance of the secondary sex characteristics. The mean values±SE of ETR_max_ for female and male individuals were 101 ± 3 and 102 ± 3 μmol m^−2^ s^−1^, respectively. Value of Φ_PPFsat_ was increased by fertilization (0.250 ± 0.10) compared with non-fertilized plants (0.199 ± 0.10) (Table 2; Figures 2A,B). Significant interaction between the sampling date (time) and fertilization treatment indicates that the differences between the fertilization treatments were modified by seasonal changes of ETR_max_, Φ_PPFsat_, and NPQ_345_ (Figure 2). Interestingly, females had the significantly higher values of F_v_/F_m_ compared with males (Table 2, Figure 3A). F_v_/F_m_ increased from September 2013 to the first appearance of cones in September 2014. Then, this parameter stabilized at the higher in female and lower level in male needles (Figure 3A). In September 2013, F_v_/F_m_ attained the lowest value of 0.747 ± 0.012 in needles of non-fertilized male individuals, and in September 2014 the highest value of 0.862 ± 0.003 in needles of fertilized female plants. The effect of time and the interactions: time x fertilization and time x sex were significant indicating that seasonal changes modified effects of the experimental treatments (Table 2). Significant differences between the sexes within the same date of fluorescence measurements were found in September 2013, December 2014, and in March 2015 (Figure 3A). F_v_/F_m_ < 0.8 indicating photoinhibition or PSII down-regulation was more often observed in non-fertilized males compared to females. When data from the whole experiment were pooled, F_v_/F_m_ of non-fertilized males was 0.788 ± 0.006, non-fertilized females 0.813 ± 0.004, fertilized males 0.804 ± 0.003, and fertilized females 0.827 ± 0.004.

**Table 2:** ANOVA results for chlorophyll *a* fluorescence light curves: ETR_max_, apparent maximum electron transport rate; Φ_PPFsat_, quantum yield of PSII photochemistry at the saturation value of photosynthetic photon flux; F_v_/F_m_, maximum quantum yield of PSII photochemistry; NPQ_345_, non-photochemical quenching of fluorescence at PPF = 345 μmol m^−2^ s^−1^; PPF_sat_, saturation photosynthetic photon flux corresponding to maximum electron transport rate.

**Parameter**	**Effect**	**DF**	**F Ratio**	***P***
ETR_max_	Sex	1	0.109	0.742
	**Fertilization**	1	92.888	<**0.0001**
	Sex^*^Fertilization	1	0.029	0.866
	**Time**	7	12.340	<**0.0001**
	Time^*^Sex	7	0.949	0.470
	Time^*^Fertilization	7	11.015	<**0.0001**
	Time^*^Sex^*^Fertilization	7	0.362	0.923
Φ_PPFsat_	Sex	1	0.632	0.428
	**Fertilization**	1	40.758	<**0.0001**
	Sex^*^Fertilization	1	2.294	0.132
	**Time**	7	15.094	<**0.0001**
	Time^*^Sex	7	1.293	0.257
	Time^*^Fertilization	7	5.808	<**0.0001**
	Time^*^Sex^*^Fertilization	7	0.997	0.436
F_v_/F_m_	**Sex**	1	19.308	<**0.0001**
	**Fertilization**	1	5.172	**0.024**
	Sex^*^Fertilization	1	0.526	0.470
	**Time**	7	11.213	<**0.0001**
	Time^*^Sex	7	1.235	0.287
	Time^*^Fertilization	7	0.701	0.671
	Time^*^Sex^*^Fertilization	7	0.302	0.952
NPQ_345_	Sex	1	0.674	0.413
	**Fertilization**	1	92.580	<**0.0001**
	Sex^*^Fertilization	1	0.176	0.675
	**Time**	7	15.260	<**0.0001**
	Time^*^Sex	7	0.891	0.515
	Time^*^Fertilization	7	3.303	**0.003**
	Time^*^Sex^*^Fertilization	7	0.639	0.723
PPF_sat_	Sex	1	0.242	0.624
	**Fertilization**	1	16.829	<**0.0001**
	Sex^*^Fertilization	1	2.407	0.123
	**Time**	7	13.904	<**0.0001**
	Time^*^Sex	7	0.502	0.832
	Time^*^Fertilization	7	1.835	0.084
	Time^*^Sex^*^Fertilization	7	0.322	0.943

*Time, fertilization group, and sex were used as the sources of fixed effects within ANOVA and block as a random effect. DF, degree of freedom; F, value of Snedecor's function; P, probability*.

### Needle structure, photosynthetic pigments concentrations, and absorptance

**Figure 4:**
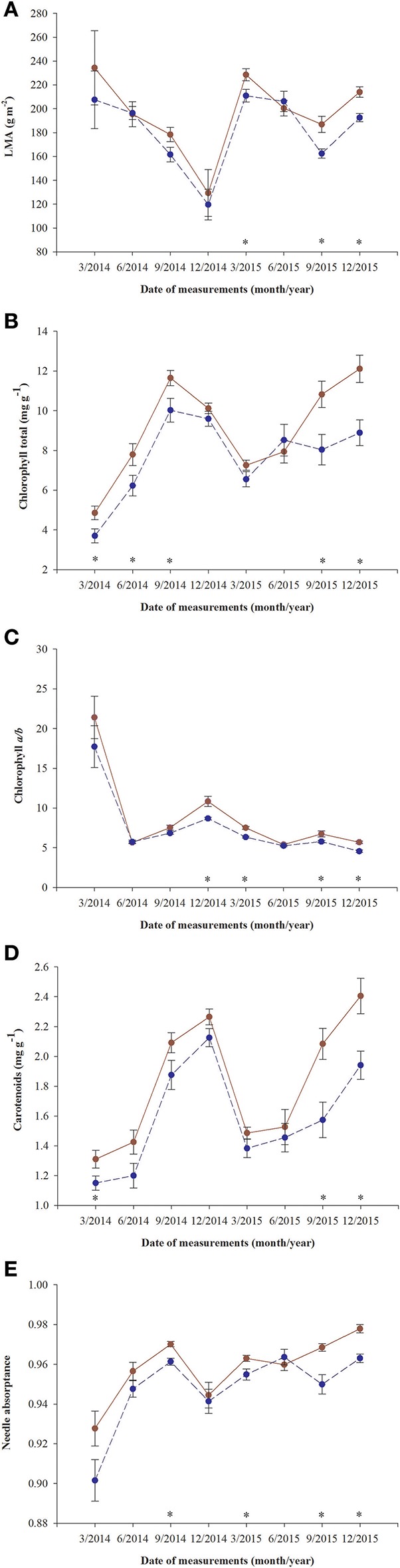
Changes in time of leaf parameters in needles of *T. baccata* individuals growing with (brown solid line) or without fertilization (dark blue dotted line): **(A)** Leaf mass-to-area ratio (LMA); **(B)** Total chlorophyll concentration (mg g^−1^); **(C)** Chlorophyll *a/b* ratio; **(D)** Carotenoids concentration (mg g^−1^); and **(E)** Needle absorptance. Data are means with standard errors (SE, *n* = 12). Asterisks indicate statistical differences between means with *P* < 0.05.

A significant decrease in LMA was detected in January 2015 in both fertilized and non-fertilized individuals (Figure 4A). Fertilized plants had higher LMA (198.5 ± 5.3 g m^−2^) than non-fertilized ones (183.7 ± 4.3). Total needle chlorophyll concentration changed over time (Figure 4B). The lowest values were observed in March 2014 (4.3 ± 0.3 mg g^−1^), the highest in September (10.8 ± 0.4). In fertilized plants total chlorophyll concentration was on average 9.1 ± 0.3 and in non-fertilized ones 7.6 ± 0.3 (Table 3). The trend of seasonal changes in carotenoids concentration was similar to that of total chlorophyll concentration (Figure 4D). There were higher carotenoids concentration in fertilized (1.81 ± 0.04 mg g^−1^) compared with non-fertilized individuals (1.59 ± 0.05) (Table 3, Figure 4D). The time-course of needle absorptance (α) increased till September 2014 and after a remarkable depletion in January 2015 it was stable except for a small decline in non-fertilized individuals in September 2015 (Figure 4E). Plants differed significantly between fertilization treatments showing higher α in needles of fertilized (0.960 ± 0.002) compared with non-fertilized ones (0.947 ± 0.002). There was also a significant interaction between time and fertilization in LMA, needle absorptance, and photosynthetic pigments concentrations (including chlorophyll *a/b* ratio; Table 3). Fertilized individuals generally had higher values of analyzed parameters than non-fertilized ones (Figure 4).

**Table 3:** ANOVA results for leaf mass-to-area ratio (LMA, g m^−2^), total chlorophyll concentration (mg g^−1^), chlorophyll *a* to *b* ratio, carotenoids (mg g^−1^), needle absorptance (α).

**Parameter**	**Effect**	**DF**	**F Ratio**	***P***
LMA	Sex	1	0.328	0.567
	**Fertilization**	1	5.655	**0.019**
	Sex^*^Fertilization	1	0.137	0.712
	**Time**	7	12.595	<**0.0001**
	Time^*^Sex	7	0.827	0.566
	Time^*^Fertilization	7	0.291	0.957
	Time^*^Sex^*^Fertilization	7	0.967	0.457
Total chlorophyll	Sex	1	0.714	0.400
	**Fertilization**	1	32.406	<**0.0001**
	Sex^*^Fertilization	1	1.234	0.268
	**Time**	7	34.990	<**0.0001**
	Time^*^Sex	7	1.538	0.158
	Time^*^Fertilization	7	1.794	0.092
	Time^*^Sex^*^Fertilization	7	0.450	0.869
Chlorophyll *a/b*	Sex	1	0.952	0.331
	**Fertilization**	1	7.266	**0.008**
	Sex^*^Fertilization	1	1.716	0.192
	**Time**	7	54.926	<**0.0001**
	Time^*^Sex	7	2.395	**0.024**
	Time^*^Fertilization	7	0.854	0.545
	Time^*^Sex^*^Fertilization	**7**	2.366	**0.025**
Carotenoids	Sex	1	1.176	0.280
	**Fertilization**	1	30.997	<**0.0001**
	Sex^*^Fertilization	1	1.604	0.207
	**Time**	7	41.836	<**0.0001**
	Time^*^Sex	7	1.477	0.179
	Time^*^Fertilization	7	1.860	0.080
	Time^*^Sex^*^Fertilization	7	0.525	0.814
Needle absorptance (α)	Sex	1	0.370	0.544
	**Fertilization**	1	24.731	<**0.0001**
	Sex^*^Fertilization	1	0.054	0.817
	**Time**	7	28.353	<**0.0001**
	Time^*^Sex	7	2.545	**0.017**
	Time^*^Fertilization	7	1.424	0.199
	Time^*^Sex^*^Fertilization	7	0.278	0.962

*Time, fertilization group, and sex were used as sources of fixed effects within ANOVA with block as source of random effect. DF, degree of freedom; F, value of Snedecor's function; P, probability*.

No significant differences between sexes were found in the variables measured (Table 3, Figure 4), although there was a significant interaction between sex and time for chlorophyll *a/b* ratio and needle absorptance (Table 3, Figures 3B,C).

### Enzyme activities

**Figure 5:**
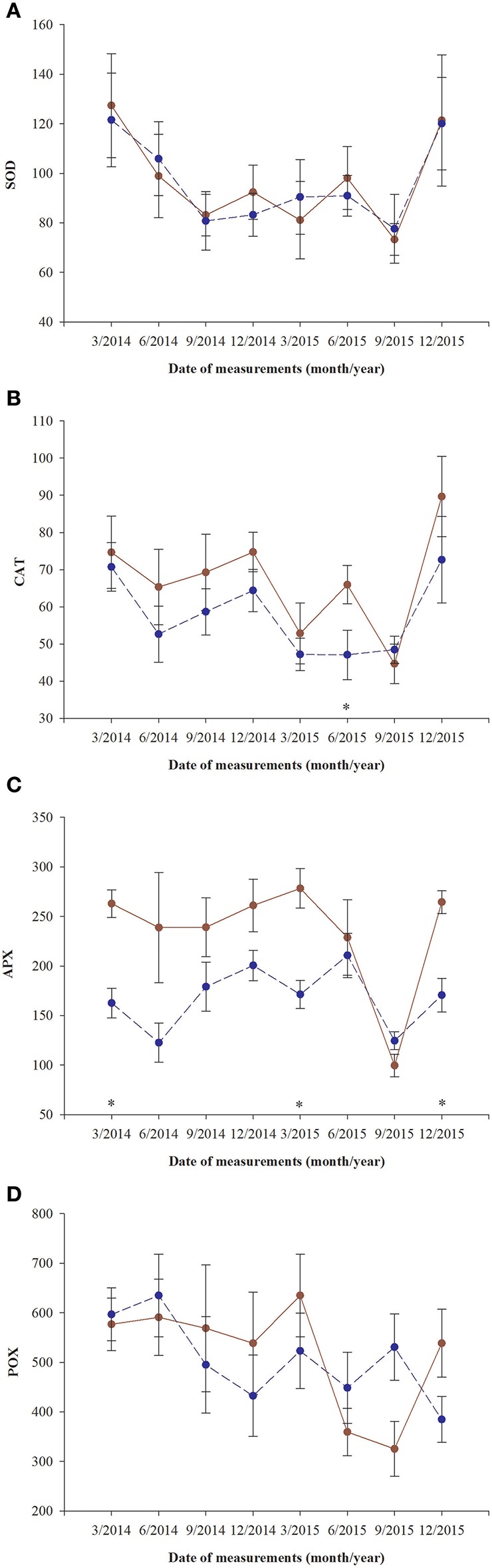
Changes with time in antioxidant systems in needles of *T. baccata* individuals growing with (brown solid line) or without fertilization (dark blue dotted line) in total activities of: **(A)** superoxide dismutase (U mg. protein^−1^, SOD); **(B)** catalase (1 mmol H_2_O_2_ min^−1^ mg. protein^−1^, CAT); **(C)** ascorbate peroxidase (1 nmol ASA min^−1^ mg. protein^−1^, APX); and **(D)** guaiacol peroxidase (nkat min^−1^ mg. protein^−1^, POX). Data are means with standard errors (SE, *n* = 12). Asterisks indicates statistical differences between means with *P* < 0.05.

Superoxidase dismutase (SOD) activity was neither influenced by fertilization nor by sex, however underwent significant changes over time (Table 4, Figure 5). The mean values of SOD for fertilized and non-fertilized individuals were 96.9 ± 5.8 U mg protein^−1^ and 96.3 ± 5.1 and for male and female individuals 101.7 ± 6.1 and 91.6 ± 4.7, respectively. The activity of catalase (CAT) and ascorbate peroxidase (APX) changed significantly with time and were generally higher in the fertilized (67.1 ± 3.2 mmol H_2_O_2_ min^−1^ mg. protein^−1^ and 234.1 ± 11.4 nmol ASA min^−1^ mg. protein^−1^) compared to non-fertilized plants (57.7 ± 2.6 and 177.1 ± 10.9) except for September 2015 when non-fertilized plants had the higher level of activity of both enzymes (Table 4, Figure 5). There was a significant interaction between time and fertilization in the case of APX but not between time and sex (Table 4, Figure 3D). APX activities increased up to March 2015 and abruptly decreased in September 2015 (Figure 3D). Guaiacol peroxidase (POX) activities were influenced by sex and were higher in females (540.7 ± 30.7 nkat min^−1^ mg. protein^−1^) compared with males (451.8 ± 22.8) except for June 2014 and March 2015 (Figure 3E, Supplementary file—Table 1).

**Table 4:** ANOVA results for total enzyme activities of: CAT, catalase; APX, ascorbate peroxidase; POX, guaiacol peroxidase; SOD, superoxide dismutase.

**Parameter**	**Effect**	**DF**	**F Ratio**	***P***
CAT	Sex	1	0.952	0.331
	**Fertilization**	1	6.052	**0.015**
	Sex^*^Fertilization	1	0.011	0.918
	**Time**	7	4.697	<**0.0001**
	Time^*^Sex	7	1.272	0.267
	Time^*^Fertilization	7	0.462	0.861
	Time^*^Sex^*^Fertilization	7	0.330	0.939
APX	Sex	1	0.661	0.417
	**Fertilization**	1	30.850	<**0.0001**
	Sex^*^Fertilization	1	0.093	0.760
	**Time**	7	5.093	<**0.0001**
	Time^*^Sex	7	2.459	**0.020**
	Time^*^Fertilization	7	2.138	**0.043**
	Time^*^Sex^*^Fertilization	7	1.249	0.279
POX	**Sex**	1	4.772	**0.030**
	Fertilization	1	0.674	0.413
	Sex^*^Fertilization	1	0.573	0.450
	Time	7	1.711	0.110
	Time^*^Sex	7	1.008	0.428
	Time^*^Fertilization	7	1.894	0.074
	Time^*^Sex^*^Fertilization	7	1.044	0.402
SOD	Sex	1	1.807	0.181
	Fertilization	1	0.007	0.932
	Sex^*^Fertilization	1	1.567	0.213
	**Time**	7	2.828	**0.008**
	Time^*^Sex	7	1.595	0.140
	Time^*^Fertilization	7	0.103	0.998
	Time^*^Sex^*^Fertilization	7	0.358	0.925

*Time, fertilization group, and sex were used as sources of fixed effect in the ANOVA with block as a source of random effect. DF, degree of freedom; F, value of Snedecor's function; P, probability*.

## Discussion

The results of our study show that the effects of fertilization treatment and seasonal changes on photochemical parameters, photosynthetic pigments' concentrations, and antioxidant enzymes activity overrode between-sexes differences in reproductive efforts except for F_v_/F_m_ and guaiacol peroxidase activity (POX). Additionally, the significant interaction between time and sex effects indicates that an effect of sex on the chlorophyll *a/b* ratio and needle absorptance was modified by seasonal changes. Therefore, we conclude that the greater reproductive efforts of female *T*. *baccata* individuals were not at the cost of their photosynthetic capacity expressed as ETR_max_ and Φ_PPFsat_. Our results are partially consistent with those of Mitchell (1998) who found that *T*. *baccata* females and males did not differ in photosynthetic leaf traits. Moreover, they are in agreement with the photosynthetic responses of *Silene latifolia* genders growing under low levels of light, water, and nutrients (Gehring and Monson, 1994). However, our findings are not consistent with earlier results of other authors who indicate that females show greater reproductive efforts than males at the expense of photosynthetic performance (Gehring and Monson, 1994; Obeso, 2002; Wheelwright and Logan, 2004; Xu et al., 2008).

Contrary to our first hypothesis, higher F_v_/F_m_ in needles of female individuals indicates that they had a potentially higher capacity of light absorption, but it also means that they had been adapted to the low light environment and were thus more threatened by photoinhibition when exposed to high light compared with males (Maxwell and Johnson, 2000; Wyka et al., 2007). Our results are in agreement with the earlier findings of Xu et al. (2008) and Morales et al. (2016) suggesting that females are generally more sensitive to photoinhibition than males.

In our experiment, the male and female individuals were grown in the same light conditions, therefore lower values of F_v_/F_m_ in *T*. *baccata* males compared with females suggest that males are evolutionarily adapted to a higher light environment. Females of this species are usually smaller and grow for longer under the shade of neighboring trees' crowns compared with males who are tall and better illuminated (Iszkuło et al., 2009). Moreover, females adapted to lower light environments might develop a different strategy of photoprotection than males. In our study, the PSII down-regulation in males did not significantly decrease their photosynthetic capacity compared with females, thus we state that it plays a photoprotective role as has been found in other conifer species (Adams et al., 2004). These results were confirmed by the enzyme activity data since the activity of POX was higher in female than in male needles, suggesting that females had a more efficient mechanism for scavenging free radicals (Sharma et al., 2012). This confirms our third hypothesis. In contrast to our results Mitchell (1998) did not find differences in F_v_/F_m_ between *T*. *baccata* males and females growing in sun. Moreover, Juvany et al. (2014) also did not find differences in F_v_/F_m_ between *Pistacia lentiscus* sexes, but NPQ values in female were higher than in male leaves indicating better photoprotection in female leaves. However, Xu et al. (2008) found higher F_v_/F_m_ in male than in female *Populus cathayana* leaves only when they were exposed to drought. The differences between sexes in photochemistry and ability for photoprotection found in our study suggest that results depend on species and conditions of growth.

All of the mechanisms which help avoid photodamage, and which repair photodamage, have the potential to decrease the net CO_2_ assimilation rates and growth, and can be regarded as costs of photoinhibition (Raven, 2011). Mechanisms of photoprotection have been developed in the course of evolution of oxygenic photosynthesis, which ensures flexible light energy utilization in a variety of plant habitats (Ruban, 2015). In our experiment conducted under artificial shading, there was no durable or severe photoinhibition even in winter, but was replaced by PSII down-regulation, especially in non-fertilized males. Although the presented results do not allow us to evaluate fully the differences between *T*. *baccata* sexes in costs of photoprotection, the higher activity of POX and APX in female needles suggests that *T*. *baccata* females made greater efforts for protection against ROS than males. In addition, antioxidant enzymes also have an antagonistic effect on auxin activity (Sofo et al., 2004). This can explain to some extent the slower growth of *T*. *baccata* females compared with males.

Our findings corroborate the earlier results which indicate that fertilized young plants show higher photosynthesis and they are less affected by photoinhibition than non-fertilized ones (Nunes et al., 1993). Fertilization increased photosynthetic capacity of *T*. *baccata* expressed as ETR_max_ and Φ_PPFsat_. This result is consistent with the general trend in conifers that indicates nitrogen concentration is positively correlated with photosynthetic capacity (Gough et al., 2004a). However, in the long term, a positive fertilization effect on photosynthesis can disappear as in *Pinus taeda* L. (Gough et al., 2004b). Improved photosynthetic capacity of fertilized conifers such as *P. taeda, Pinus sylvestris*, and *Abies alba* has been shown to be due to more efficient and enhanced capacity of electron transport as it was in our study (Kellomäki and Wang, 1997; Lavigne et al., 2001) and/or greater carboxylation capacity (Kellomäki and Wang, 1997; Maier et al., 2002; Robakowski et al., 2003).

In our experiment, non-fertilized female and male *T*. *baccata* individuals displayed lower photochemical parameters, lower total chlorophyll and carotenoid concentrations, and higher activities of antioxidant enzymes (POX, APX, SOD) than fertilized individuals (hypothesis 4). This is consistent with earlier findings indicating that Mg-deficiency decreased concentrations of photosynthetic pigments and increased the activities of antioxidants (Laing et al., 2000; Tewari et al., 2006; Chou et al., 2011; Tang et al., 2012). In our study, under nutrient deficiency, a reduction of ETR resulted from a decrease in Φ_PSII_, and to a lesser extent to a decrease in α which was directly dependent on needle chlorophyll concentration. When plants are stressed by nutrient deficiency or excessive light, the acceptor side of PSII is overreduced, and the plants produce ROS near PSII. A delay of electron transport between PSII and PSI is due to their separation from the stroma thylakoids and the grana, respectively. This delay of electron transport promotes ROS generation such as the free radicals O^·−2^ and OH^·−^ and non-radicals like H_2_O_2_ and ^1^O_2_ (Das and Roychoudhury, 2014; Yoshioka-Nishimura, 2016). Our results have shown that non-fertilized *T*. *baccata* individuals having lower ETR_max_ at lower values of PPF_sat_, lower F_v_/F_m_ and higher NPQ_345_ along with higher activity of CAT, APX, and POX compared with fertilized individuals were more sensitive to photoinhibition induced by nutrients deficiency and low winter temperatures in December. This indicates that the antioxidant system is active and maintains the electron flow balance (Adams et al., 2004; Huseynova, 2012). Higher NPQ_345_ has also indicated that in non-fertilized individuals the higher amount of energy was dissipated as heat than in fertilized individuals, and the cost of this photoprotective mechanism, expressed as energy losses, can be greater under nutrient deficiency.

We did not find any significant interaction between the gender and fertilization. This indicates that both sexes reacted in a similar way to the stress conditions caused by nutrients deficiency. Male and female individuals of *T*. *baccata* acclimated to nutrients deficiency by lowering photosynthetic capacity and needle pigments concentrations. Total chlorophyll and carotenoids concentrations in needles changed seasonally and were lower without fertilization, but were independent of sex. However, in contrast to our results, Zarek (2015) found that there were higher concentrations of total chlorophyll and carotenoids in needles of adult female trees of *T*. *baccata* compared with males in autumn and in winter. This discrepancy may result from the fact that we used young individuals, and not mature trees. This confirms the conclusions from earlier research that long-term studies are needed to elucidate the responses of the sexes to multiple stressors (Suzuki et al., 2014; Juvany and Munné-Bosch, 2015; Retuerto et al., 2018). Female costs, in terms of carbon, may be at least partially compensated by increasing photosynthetic rates or by the photosynthesis of non-matured cones or fruits. We measured chlorophyll *a* fluorescence of a cross section of green *T*. *baccata* arils. The values of F_v_/F_m_ ranging from 0.78 to 0.80 are lower than the optimum value of 0.84, but this suggests that green arils are able to photosynthesize and compensate to some extent the female reproductive effort. However, photosynthesis by green arils cannot cover construction and maintenance of non-photosynthetic seed and red arils. In a similar way, photosynthetic activity of reproductive structures in the dioecious Neotropical tree *Ocotea tenera* was insufficient to pay the high costs of reproduction borne by females (Wheelwright and Logan, 2004).

In conclusion, our results indicate that females of *T*. *baccata* have similar photosynthetic capacity to males and the higher reproductive efforts of females are not at the expense of their photosynthetic capacity. The maintenance of photosynthetic capacity and higher reproductive efforts of *T*. *baccata* females were at the cost of antioxidants production. The higher reproductive efforts of females did not decrease their photosynthetic capacity suggesting that the between-sexes differences in F_v_/F_m_ and antioxidants activity found in our study resulted from the adaptation of females and males to different growth environments.

## Author contributions

Study conception and design: PR, EP-K, and GI; acquisition of data: PR, EP-K, ER, and MR; analysis, interpretation of data, and drafting of manuscript: PR, ER, EP-K, and GI; critical revision: PT and Z-PY; final version: PR, EP-K, ER, PT, Z-PY, and GI.

### Conflict of interest statement

The authors declare that the research was conducted in the absence of any commercial or financial relationships that could be construed as a potential conflict of interest.

## Abbreviations

ETR: apparent electron transport rate (μmol m^−2^ s^−1^)
ETR_max_: maximum value of ETR
F_v_/F_m_: maximum quantum yield of photosystem II photochemistry
F_m_: maximum fluorescence yield
F'_m_: maximum fluorescence in the light
F_0_: minimum fluorescence yield
F_s_: steady-state fluorescence
NPQ: non-photochemical quenching of fluorescence
PPF: photosynthetic photon flux (μmol m^−2^ s^−1^)
PPF_sat_: saturating level of photosynthetic photon flux
PSII: photosystem II
Φ_PSII_: quantum yield of PSII photochemistry
Φ_PPFsat_: quantum yield of PSII photochemistry at the saturation level of PPF
SOD: superoxide dismutase
POX: guaiacol peroxidase
CAT: catalase
APX: ascorbate peroxidase.
