# Supplementary material for: Photochemistry and Antioxidative Capacity of Female and Male Taxus baccata L. Acclimated to Different Nutritional Environments
Source: Front Plant Sci. 2018 Jun 5;9:742. doi: 10.3389/fpls.2018.00742 (PMC5996056; doi:10.3389/fpls.2018.00742)
Supplement: Supplementary file 1 [file Data_Sheet_1.doc]

**Supplementary file.**

**Authors:** Piotr Robakowski, Emilia Pers-Kamczyc, Ewelina Ratajczak, Peter A. Thomas, Zi-Piao Ye, Mariola Rabska, Grzegorz Iszkuło

**Title of manuscript**: Photochemistry and Antioxidative Capacity of Female and Male *Taxus baccata* L. Acclimated to Different Nutritional Environments

Table 1. Changes with time in analysed parameters in needles of *T. baccata* male and female individuals growing with or without fertilization. Data are means with standard errors (SE, *n* = 12).

ETR_max_- apparent maximum electron transport rate, Φ_PPFsat_ - quantum yield of PSII photochemistry at the saturation value of photosynthetic photon flux, F_v_/F_m_ - maximum quantum yield of PSII photochemistry, NPQ_345_ - non-photochemical quenching of fluorescence at PPF = 345 µmol m^-2^ s^-1^, PPF_sat_ - saturation photosynthetic photon flux corresponding to maximum electron transport rate, LMA - leaf mass-to-area ratio, CAT -catalase (mM H_2_O_2_ min^-1^mg. protein ^-1^), APX - ascorbate peroxidase (1 nM ASA min^-1^ mg protein), POX - guaiacol peroxidase (nkat min^-1^mg. protein ^-1^), SOD - superoxidase dismutase (100 mg. protein^-1^)

| **Parameter** | **Year** | **Month** | **Fertilized** | | | | **Non-Fertilized** | | | |
| --- | --- | --- | --- | --- | --- | --- | --- | --- | --- | --- |
|  |  |  | **Female** | | **Male** | | **Female** | | **Male** | |
|  |  |  | **Mean** | **SEM** | **Mean** | **SEM** | **Mean** | **SEM** | **Mean** | **SEM** |
| ETR_max_ | 2014 | 3 | 120.635 | 9.525 | 115.232 | 12.905 | 88.693 | 6.150 | 84.410 | 7.267 |
|  | 2014 | 6 | 100.436 | 3.290 | 99.795 | 9.570 | 77.379 | 4.429 | 81.530 | 8.018 |
|  | 2014 | 9 | 140.517 | 6.247 | 157.085 | 4.957 | 98.475 | 7.274 | 105.857 | 5.540 |
|  | 2014 | 12 | 128.218 | 17.295 | 124.689 | 12.753 | 83.541 | 6.827 | 94.975 | 5.493 |
|  | 2015 | 3 | 118.802 | 1.451 | 113.316 | 7.414 | 103.127 | 4.525 | 84.978 | 5.721 |
|  | 2015 | 6 | 117.019 | 5.933 | 81.882 | 7.000 | 78.685 | 7.984 | 81.882 | 7.000 |
|  | 2015 | 9 | 126.667 | 14.892 | 106.724 | 5.646 | 66.985 | 5.874 | 76.686 | 3.656 |
|  | 2015 | 12 | 97.447 | 5.859 | 92.435 | 8.219 | 52.642 | 5.777 | 58.225 | 8.095 |
|  |  |  |  |  |  |  |  |  |  |  |
| Φ_PPFsat_ | 2014 | 3 | 0.219 | 0.017 | 0.216 | 0.018 | 0.185 | 0.016 | 0.215 | 0.019 |
|  | 2014 | 6 | 0.212 | 0.010 | 0.220 | 0.020 | 0.171 | 0.016 | 0.203 | 0.012 |
|  | 2014 | 9 | 0.265 | 0.016 | 0.273 | 0.009 | 0.178 | 0.015 | 0.217 | 0.026 |
|  | 2014 | 12 | 0.307 | 0.030 | 0.260 | 0.017 | 0.185 | 0.023 | 0.225 | 0.022 |
|  | 2015 | 3 | 0.312 | 0.007 | 0.301 | 0.010 | 0.298 | 0.015 | 0.259 | 0.016 |
|  | 2015 | 6 | 0.206 | 0.018 | 0.220 | 0.022 | 0.164 | 0.018 | 0.169 | 0.023 |
|  | 2015 | 9 | 0.244 | 0.023 | 0.280 | 0.013 | 0.191 | 0.024 | 0.207 | 0.009 |
|  | 2015 | 12 | 0.247 | 0.016 | 0.213 | 0.012 | 0.154 | 0.015 | 0.140 | 0.014 |
|  |  |  |  |  |  |  |  |  |  |  |
| F_v_/F_M_ | 2014 | 3 | 0.789 | 0.014 | 0.786 | 0.017 | 0.787 | 0.011 | 0.769 | 0.017 |
|  | 2014 | 6 | 0.819 | 0.013 | 0.809 | 0.009 | 0.815 | 0.017 | 0.803 | 0.009 |
|  | 2014 | 9 | 0.862 | 0.003 | 0.851 | 0.003 | 0.824 | 0.011 | 0.840 | 0.004 |
|  | 2014 | 12 | 0.820 | 0.010 | 0.807 | 0.007 | 0.783 | 0.011 | 0.780 | 0.012 |
|  | 2015 | 3 | 0.821 | 0.011 | 0.808 | 0.006 | 0.793 | 0.005 | 0.786 | 0.009 |
|  | 2015 | 6 | 0.825 | 0.007 | 0.834 | 0.003 | 0.825 | 0.012 | 0.826 | 0.008 |
|  | 2015 | 9 | 0.838 | 0.012 | 0.816 | 0.009 | 0.811 | 0.012 | 0.802 | 0.017 |
|  | 2015 | 12 | 0.842 | 0.006 | 0.819 | 0.011 | 0.816 | 0.013 | 0.790 | 0.020 |
|  |  |  |  |  |  |  |  |  |  |  |
| NPQ_345_ | 2014 | 3 | 1.232 | 0.186 | 1.181 | 0.118 | 2.012 | 0.256 | 2.084 | 0.269 |
|  | 2014 | 6 | 1.596 | 0.138 | 1.480 | 0.252 | 2.113 | 0.150 | 1.917 | 0.171 |
|  | 2014 | 9 | 1.132 | 0.124 | 0.777 | 0.161 | 2.169 | 0.223 | 1.893 | 0.137 |
|  | 2014 | 12 | 1.296 | 0.230 | 1.370 | 0.172 | 2.154 | 0.274 | 1.975 | 0.213 |
|  | 2015 | 3 | 1.163 | 0.099 | 1.044 | 0.102 | 1.255 | 0.049 | 1.638 | 0.163 |
|  | 2015 | 6 | 1.972 | 0.245 | 1.655 | 0.257 | 2.038 | 0.220 | 1.819 | 0.185 |
|  | 2015 | 9 | 1.031 | 0.203 | 0.991 | 0.144 | 2.331 | 0.241 | 2.203 | 0.137 |
|  | 2015 | 12 | 2.196 | 0.294 | 2.247 | 0.319 | 3.441 | 0.288 | 2.808 | 0.348 |
|  |  |  |  |  |  |  |  |  |  |  |
| PPF_sat_ | 2014 | 3 | 1181.880 | 80.493 | 1130.942 | 69.737 | 1056.841 | 85.262 | 883.451 | 48.772 |
|  | 2014 | 6 | 961.505 | 31.132 | 950.968 | 64.653 | 930.279 | 76.163 | 840.341 | 55.092 |
|  | 2014 | 9 | 1140.208 | 30.983 | 1226.568 | 57.183 | 1093.937 | 52.105 | 1069.135 | 40.217 |
|  | 2014 | 12 | 902.112 | 74.171 | 1044.672 | 96.081 | 994.037 | 114.663 | 916.207 | 123.870 |
|  | 2015 | 3 | 799.999 | 13.978 | 811.165 | 36.695 | 748.649 | 16.306 | 706.586 | 26.804 |
|  | 2015 | 6 | 1040.903 | 68.582 | 1013.015 | 50.772 | 1004.132 | 125.622 | 1010.492 | 107.586 |
|  | 2015 | 9 | 1057.059 | 68.198 | 1039.638 | 85.982 | 757.457 | 98.952 | 777.048 | 52.162 |
|  | 2015 | 12 | 801.468 | 29.846 | 844.347 | 51.880 | 707.959 | 40.425 | 730.406 | 46.372 |
|  |  |  |  |  |  |  |  |  |  |  |
| LMA | 2014 | 3 | 219.297 | 32.971 | 249.465 | 55.534 | 228.975 | 45.795 | 186.303 | 17.253 |
|  | 2014 | 6 | 215.284 | 12.013 | 175.729 | 13.421 | 204.896 | 7.828 | 187.960 | 6.334 |
|  | 2014 | 9 | 184.676 | 6.391 | 172.150 | 9.997 | 172.977 | 5.042 | 150.239 | 9.389 |
|  | 2014 | 12 | 132.837 | 31.499 | 125.881 | 26.829 | 97.184 | 2.284 | 142.083 | 22.909 |
|  | 2015 | 3 | 231.113 | 7.780 | 226.017 | 7.268 | 214.067 | 5.885 | 207.933 | 9.131 |
|  | 2015 | 6 | 195.610 | 4.198 | 205.405 | 12.846 | 190.776 | 12.135 | 221.737 | 8.721 |
|  | 2015 | 9 | 189.362 | 9.741 | 184.565 | 10.405 | 165.181 | 5.411 | 159.787 | 6.162 |
|  | 2015 | 12 | 215.308 | 8.626 | 212.781 | 3.600 | 196.076 | 4.456 | 189.168 | 5.072 |
|  |  |  |  |  |  |  |  |  |  |  |
| Total chlorophyll concentration (mg/g) | 2014 | 3 | 5.155 | 0.520 | 4.562 | 0.465 | 3.821 | 0.512 | 3.583 | 0.514 |
|  | 2014 | 6 | 7.776 | 0.937 | 7.826 | 0.674 | 6.123 | 0.691 | 6.337 | 0.829 |
|  | 2014 | 9 | 11.767 | 0.515 | 11.533 | 0.611 | 8.940 | 0.826 | 11.117 | 0.659 |
|  | 2014 | 12 | 10.151 | 0.467 | 10.082 | 0.294 | 9.143 | 0.532 | 10.051 | 0.500 |
|  | 2015 | 3 | 7.499 | 0.460 | 7.016 | 0.223 | 6.683 | 0.373 | 6.420 | 0.713 |
|  | 2015 | 6 | 8.621 | 1.068 | 7.264 | 0.347 | 9.125 | 1.468 | 8.025 | 0.875 |
|  | 2015 | 9 | 9.752 | 1.065 | 11.893 | 0.565 | 7.334 | 1.084 | 8.751 | 1.086 |
|  | 2015 | 12 | 12.189 | 1.028 | 12.033 | 0.996 | 8.129 | 0.591 | 9.654 | 1.114 |
|  |  |  |  |  |  |  |  |  |  |  |
| Chlorophyll *a/b* | 2014 | 3 | 15.859 | 1.753 | 26.033 | 3.792 | 17.766 | 4.133 | 17.680 | 3.494 |
|  | 2014 | 6 | 5.642 | 0.135 | 5.687 | 0.127 | 5.837 | 0.218 | 5.658 | 0.361 |
|  | 2014 | 9 | 8.015 | 0.480 | 7.054 | 0.367 | 6.861 | 0.234 | 6.826 | 0.256 |
|  | 2014 | 12 | 12.046 | 0.882 | 9.622 | 0.735 | 8.856 | 0.095 | 8.517 | 0.328 |
|  | 2015 | 3 | 7.417 | 0.270 | 7.603 | 0.257 | 6.475 | 0.175 | 6.216 | 0.169 |
|  | 2015 | 6 | 5.556 | 0.174 | 5.254 | 0.259 | 5.259 | 0.114 | 5.246 | 0.258 |
|  | 2015 | 9 | 6.298 | 0.639 | 7.189 | 0.321 | 5.877 | 0.242 | 5.680 | 0.187 |
|  | 2015 | 12 | 5.817 | 0.194 | 5.574 | 0.299 | 4.479 | 0.107 | 4.653 | 0.300 |
|  |  |  |  |  |  |  |  |  |  |  |
| Carotenoids concentration (mg/g) | 2014 | 3 | 1.338 | 0.106 | 1.283 | 0.065 | 1.179 | 0.081 | 1.121 | 0.056 |
|  | 2014 | 6 | 1.397 | 0.142 | 1.453 | 0.087 | 1.180 | 0.107 | 1.219 | 0.136 |
|  | 2014 | 9 | 2.117 | 0.101 | 2.067 | 0.099 | 1.692 | 0.132 | 2.059 | 0.105 |
|  | 2014 | 12 | 2.274 | 0.082 | 2.256 | 0.073 | 2.035 | 0.083 | 2.217 | 0.076 |
|  | 2015 | 3 | 1.545 | 0.068 | 1.427 | 0.030 | 1.398 | 0.057 | 1.367 | 0.115 |
|  | 2015 | 6 | 1.548 | 0.177 | 1.362 | 0.074 | 1.593 | 0.228 | 1.471 | 0.123 |
|  | 2015 | 9 | 1.922 | 0.170 | 2.246 | 0.088 | 1.435 | 0.177 | 1.711 | 0.155 |
|  | 2015 | 12 | 2.432 | 0.189 | 2.379 | 0.160 | 1.816 | 0.112 | 2.066 | 0.144 |
|  |  |  |  |  |  |  |  |  |  |  |
| Needle absorptance (α) | 2014 | 3 | 0.936 | 0.010 | 0.918 | 0.015 | 0.915 | 0.011 | 0.885 | 0.017 |
|  | 2014 | 6 | 0.960 | 0.006 | 0.953 | 0.007 | 0.950 | 0.005 | 0.946 | 0.007 |
|  | 2014 | 9 | 0.972 | 0.002 | 0.969 | 0.002 | 0.960 | 0.003 | 0.963 | 0.002 |
|  | 2014 | 12 | 0.942 | 0.012 | 0.947 | 0.006 | 0.933 | 0.005 | 0.950 | 0.011 |
|  | 2015 | 3 | 0.964 | 0.002 | 0.962 | 0.002 | 0.957 | 0.003 | 0.953 | 0.005 |
|  | 2015 | 6 | 0.961 | 0.005 | 0.958 | 0.004 | 0.963 | 0.007 | 0.964 | 0.004 |
|  | 2015 | 9 | 0.965 | 0.003 | 0.972 | 0.002 | 0.945 | 0.009 | 0.955 | 0.003 |
|  | 2015 | 12 | 0.976 | 0.002 | 0.980 | 0.004 | 0.961 | 0.003 | 0.965 | 0.004 |
|  |  |  |  |  |  |  |  |  |  |  |

| CAT | 2014 | 3 | 70.098 | 11.078 | 71.129 | 14.595 | 73.088 | 8.861 | 79.230 | 16.906 |
| --- | --- | --- | --- | --- | --- | --- | --- | --- | --- | --- |
|  | 2014 | 6 | 48.562 | 9.615 | 82.115 | 15.886 | 41.407 | 7.108 | 63.873 | 12.240 |
|  | 2014 | 9 | 67.453 | 15.758 | 71.129 | 14.595 | 63.052 | 7.437 | 54.251 | 10.395 |
|  | 2014 | 12 | 82.568 | 8.223 | 66.911 | 5.543 | 62.969 | 4.870 | 65.831 | 10.901 |
|  | 2015 | 3 | 53.056 | 11.326 | 52.619 | 12.838 | 47.165 | 7.395 | 47.190 | 5.391 |
|  | 2015 | 6 | 71.612 | 8.290 | 60.287 | 6.119 | 48.337 | 10.737 | 45.821 | 8.903 |
|  | 2015 | 9 | 42.964 | 9.325 | 46.339 | 5.958 | 50.837 | 5.244 | 46.056 | 5.463 |
|  | 2015 | 12 | 84.313 | 13.010 | 95.018 | 18.203 | 61.583 | 9.482 | 83.742 | 21.388 |
|  |  |  |  |  |  |  |  |  |  |  |
| APX | 2014 | 3 | 268.903 | 15.585 | 237.724 | 36.848 | 139.701 | 24.367 | 257.022 | 24.469 |
|  | 2014 | 6 | 141.113 | 37.796 | 336.388 | 90.932 | 112.441 | 30.705 | 132.711 | 27.338 |
|  | 2014 | 9 | 240.514 | 50.147 | 237.724 | 36.848 | 185.347 | 49.157 | 172.907 | 16.318 |
|  | 2014 | 12 | 231.675 | 18.953 | 290.596 | 48.882 | 192.996 | 20.162 | 208.214 | 24.742 |
|  | 2015 | 3 | 283.306 | 41.090 | 273.262 | 6.468 | 152.954 | 19.765 | 189.691 | 18.621 |
|  | 2015 | 6 | 271.914 | 70.361 | 185.537 | 27.002 | 248.523 | 36.404 | 173.012 | 17.245 |
|  | 2015 | 9 | 109.006 | 19.511 | 90.091 | 11.930 | 124.433 | 10.914 | 124.562 | 15.303 |
|  | 2015 | 12 | 273.336 | 19.145 | 255.603 | 13.274 | 163.968 | 27.886 | 177.157 | 22.239 |
|  |  |  |  |  |  |  |  |  |  |  |
| POX | 2014 | 3 | 622.781 | 84.134 | 384.911 | 104.441 | 714.024 | 77.418 | 531.077 | 67.000 |
|  | 2014 | 6 | 568.159 | 137.155 | 613.691 | 83.656 | 321.591 | 71.643 | 467.776 | 67.108 |
|  | 2014 | 9 | 752.674 | 218.489 | 384.911 | 104.441 | 492.132 | 176.342 | 498.044 | 102.484 |
|  | 2014 | 12 | 630.201 | 132.504 | 446.963 | 161.994 | 543.999 | 110.120 | 320.890 | 112.506 |
|  | 2015 | 3 | 707.862 | 151.662 | 562.051 | 72.898 | 426.185 | 102.898 | 620.248 | 105.317 |
|  | 2015 | 6 | 380.530 | 78.099 | 338.166 | 61.755 | 546.987 | 119.266 | 350.193 | 69.168 |
|  | 2015 | 9 | 351.347 | 91.161 | 299.560 | 71.113 | 631.212 | 80.236 | 430.479 | 95.157 |
|  | 2015 | 12 | 629.928 | 99.422 | 447.226 | 86.040 | 330.994 | 51.663 | 438.537 | 75.333 |
|  |  |  |  |  |  |  |  |  |  |  |
| SOD | 2014 | 3 | 107.970 | 32.475 | 85.829 | 10.125 | 102.752 | 13.333 | 146.753 | 27.044 |
|  | 2014 | 6 | 71.613 | 17.362 | 126.201 | 25.488 | 90.769 | 23.782 | 121.021 | 18.044 |
|  | 2014 | 9 | 80.454 | 14.408 | 85.829 | 10.125 | 88.021 | 18.438 | 73.487 | 15.871 |
|  | 2014 | 12 | 109.250 | 16.307 | 75.529 | 12.247 | 93.884 | 13.056 | 72.562 | 10.857 |
|  | 2015 | 3 | 79.188 | 23.406 | 82.994 | 23.003 | 88.734 | 27.894 | 92.189 | 14.950 |
|  | 2015 | 6 | 83.229 | 8.889 | 112.927 | 23.391 | 85.290 | 10.748 | 96.658 | 13.113 |
|  | 2015 | 9 | 77.590 | 11.299 | 68.991 | 6.904 | 95.504 | 26.104 | 59.682 | 6.551 |
|  | 2015 | 12 | 88.038 | 16.574 | 154.689 | 48.634 | 122.751 | 22.107 | 117.370 | 32.190 |
